# Reliability of doming and toe flexion testing to quantify foot muscle strength

**DOI:** 10.1186/s13047-017-0237-y

**Published:** 2017-12-08

**Authors:** Sarah Trager Ridge, J. William Myrer, Mark T. Olsen, Kevin Jurgensmeier, A. Wayne Johnson

**Affiliations:** Department of Exercise Sciences, Brigham Young University, Provo, UT 84602 USA

**Keywords:** Intrinsic foot muscles, Strength testing, Doming/short foot exercise, Toe flexion

## Abstract

**Background:**

Quantifying the strength of the intrinsic foot muscles has been a challenge for clinicians and researchers. The reliable measurement of this strength is important in order to assess weakness, which may contribute to a variety of functional issues in the foot and lower leg, including plantar fasciitis and hallux valgus. This study reports 3 novel methods for measuring foot strength – doming (previously unmeasured), hallux flexion, and flexion of the lesser toes.

**Methods:**

Twenty-one healthy volunteers performed the strength tests during two testing sessions which occurred one to five days apart. Each participant performed each series of strength tests (doming, hallux flexion, and lesser toe flexion) four times during the first testing session (twice with each of two raters) and two times during the second testing session (once with each rater). Intra-class correlation coefficients were calculated to test for reliability for the following comparisons: between raters during the same testing session on the same day (inter-rater, intra-day, intra-session), between raters on different days (inter-rater, inter-day, inter-session), between days for the same rater (intra-rater, inter-day, inter-session), and between sessions on the same day by the same rater (intra-rater, intra-day, inter-session).

**Results:**

ICCs showed good to excellent reliability for all tests between days, raters, and sessions. Average doming strength was 99.96 ± 47.04 N. Average hallux flexion strength was 65.66 ± 24.5 N. Average lateral toe flexion was 50.96 ± 22.54 N.

**Conclusions:**

These simple tests using relatively low cost equipment can be used for research or clinical purposes. If repeated testing will be conducted on the same participant, it is suggested that the same researcher or clinician perform the testing each time for optimal reliability.

## Background

The ability to measure foot muscle strength accurately and reliably is necessary for clinicians and researchers to enable them to monitor strengthening or identify weakness [1]. Direct strength measurements of these muscles is challenging, due in part to the lack of commercially available equipment. In addition, the intrinsic foot muscles (IFM) have many of the same actions as the extrinsic lower limb muscles, therefore it can be difficult to isolate and assess the strength of only the intrinsic foot muscles. It may be more appropriate to test strength during specific movements rather than individual muscles, due to the simultaneous involvement of both intrinsic and extrinsic muscles.

In recent years, studies have shown that IFM strength can have varying effects on lower limb pathologies and balance [2]. In particular, IFM weakness or altered activation has been associated with multiple issues in the foot and lower leg including, pes cavus in patients with Charcot-Marie-Tooth disease [3], heel pain [4, 5], claw toe deformity [6, 7], hammer toe deformity [6, 8], hallux valgus [2, 9], and posteromedial shin pain [10, 11]. People with plantar fasciitis have been found to have weaker IFM [5] and/or lower IFM volume [12] than those with healthy feet. Recent research has also shown that IFM weakness has been associated with an increased risk of falls in older people due to their role in stabilizing the foot and maintaining balance [2, 13, 14].

Previous studies have used a variety of methods in an attempt to measure the strength of intrinsic foot muscles. Most studies have measured toe flexor force using hand-held dynamometry where the dynamometer was fixed stationary as the participants maximally pushed onto the dynamometer with their toes [1, 8, 15]. Other tests have included the Paper Grip Test, plantar pressure, or the Intrinsic Positive Test [2, 10, 16–19].

The Paper Grip Test and plantar pressure, combined, resulted in reliable measures of the intrinsic plantar flexor muscles (plantar pressure ICC 0.87 for lesser toes and 0.88 for hallux; no reliability calculated for the Paper Grip Test) [19], similar to those found when using hand-held dynamometry (ICC range 0.81–0.94, depending on the rater and the muscle group tested – ankle plantar flexion, lesser toe plantar flexion, or hallux plantar flexion) [1]. Another approach to measuring intrinsic foot muscles is known as the Intrinsic Positive Test. This test involves the researcher evaluating the participant’s ability to perform intrinsic foot muscle contractions where the participant extends the hallux while simultaneously flexing the lesser toes at the MTP joint and extending the interphalangeal joints [10]. This method of testing is less reliable than using the Paper Grip Test or hand-held dynamometry because it is not quantifiable [2]. Each test described here has a major drawback and, as such, the use of the test for research or repeated clinical purposes is less than ideal.

Another limitation to existing testing methods is the lack of data generated regarding strength during movements other than toe flexion. The doming exercise (also known as the short-foot exercise) is used by many clinicians to engage and strengthen the intrinsic foot muscles. To this point, no one has tried to quantify strength associated with performing this movement.

In order to measure strength during functional movements including doming, hallux flexion, and lesser toe flexion, we developed new methods of assessment using dynamometry, acknowledging the contribution of the intrinsic and extrinsic foot muscles in performing these movements. This included developing the only method that we are aware of for quantifying strength during the doming motion, as well as an alternative method of testing toe flexion strength that may be advantageous with regard to simplicity, expense, and/or reliability. The purpose of this study was to evaluate the reliability of these new methods.

## Methods

### Participants

Twenty-one volunteers participated in this study (13 males, 8 females; age: 24.2 ± 2.5 years, height: 170.5 ± 39.1 cm, weight: 75.9 ± 14.5 kg). All participants were healthy and free from foot pain or deformity at the time of the study. Participants signed a consent form approved by the university’s IRB in compliance with the Declaration of Helsinki and completed two testing sessions from one to five days apart.

### Procedures

**Fig. 1:**
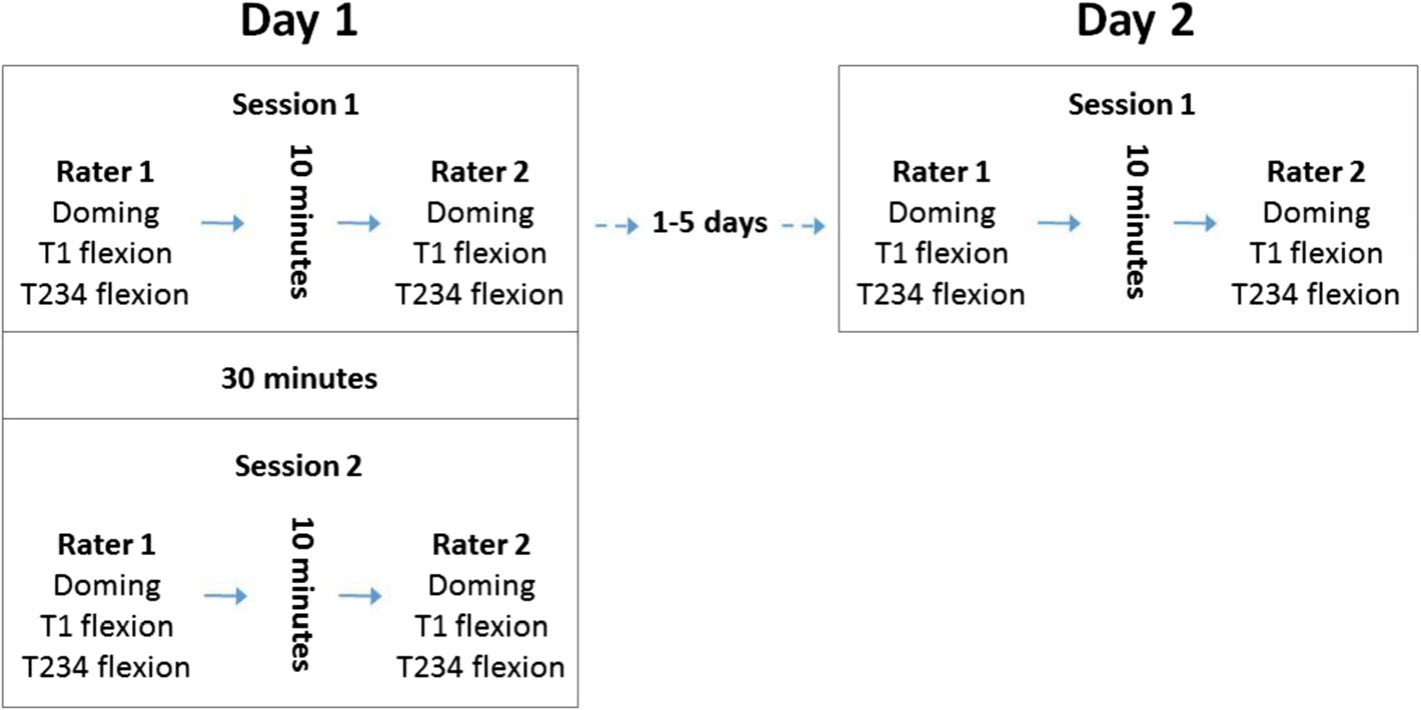
Timeline of each subject’s participation

On each of the two days of testing, participants performed a series of three foot strength tests, which were performed in a set order – doming, hallux flexion (T1), and flexion of the first three lesser toes (T234). Each series was repeated four times (two times with two different raters) on the first day and twice on the second day (one time with two different raters) (see Fig. 1 for a complete timeline of testing procedures). The second day of testing was completed one to five days after the first. A total of 10 raters performed testing throughout the study. A random pairing of raters was chosen and the same pair of raters performed the tests on a specific subject during both days of testing. All raters were trained on testing procedures and had performed testing on at least 10 practice subjects prior to performing testing for this study.

Prior to testing on the first day, each participant was shown the proper technique for the doming test. The participant practiced until the rater felt comfortable that the movements were being performed correctly, which took approximately 10 repetitions. The purpose of this was to minimize use of extrinsic foot muscles during testing.

**Fig. 2:**
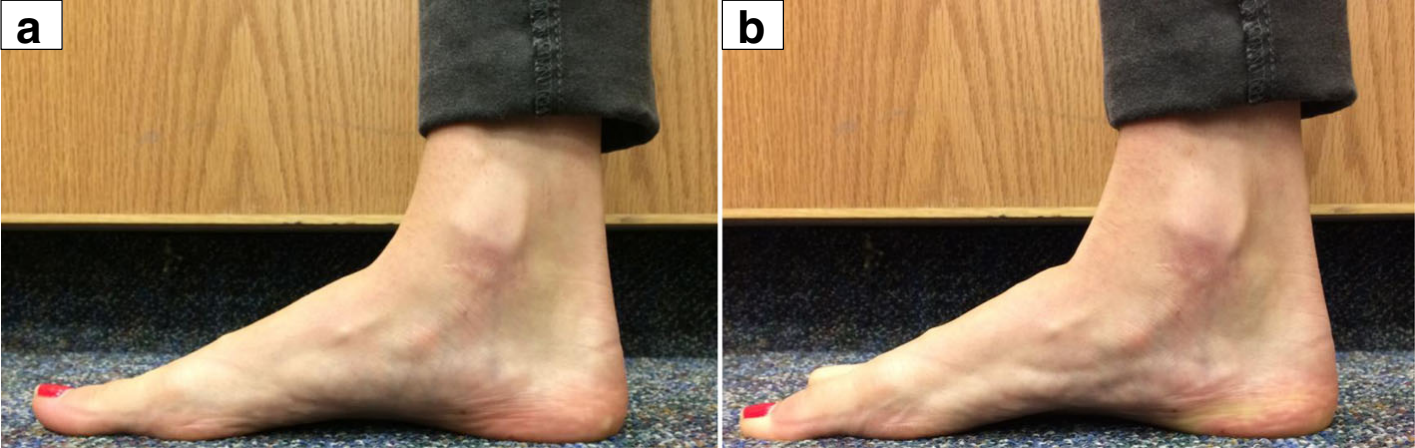
The doming (or short-foot) movement starts with the subject standing and the foot relaxed (**a**). Instructions were given to “focus on pulling the ball of the foot towards the heel without curling the toes or raising the ball of the foot off of the ground”. This results in a “shortened” foot and raised medial longitudinal arch (**b**)

**Fig. 3:**
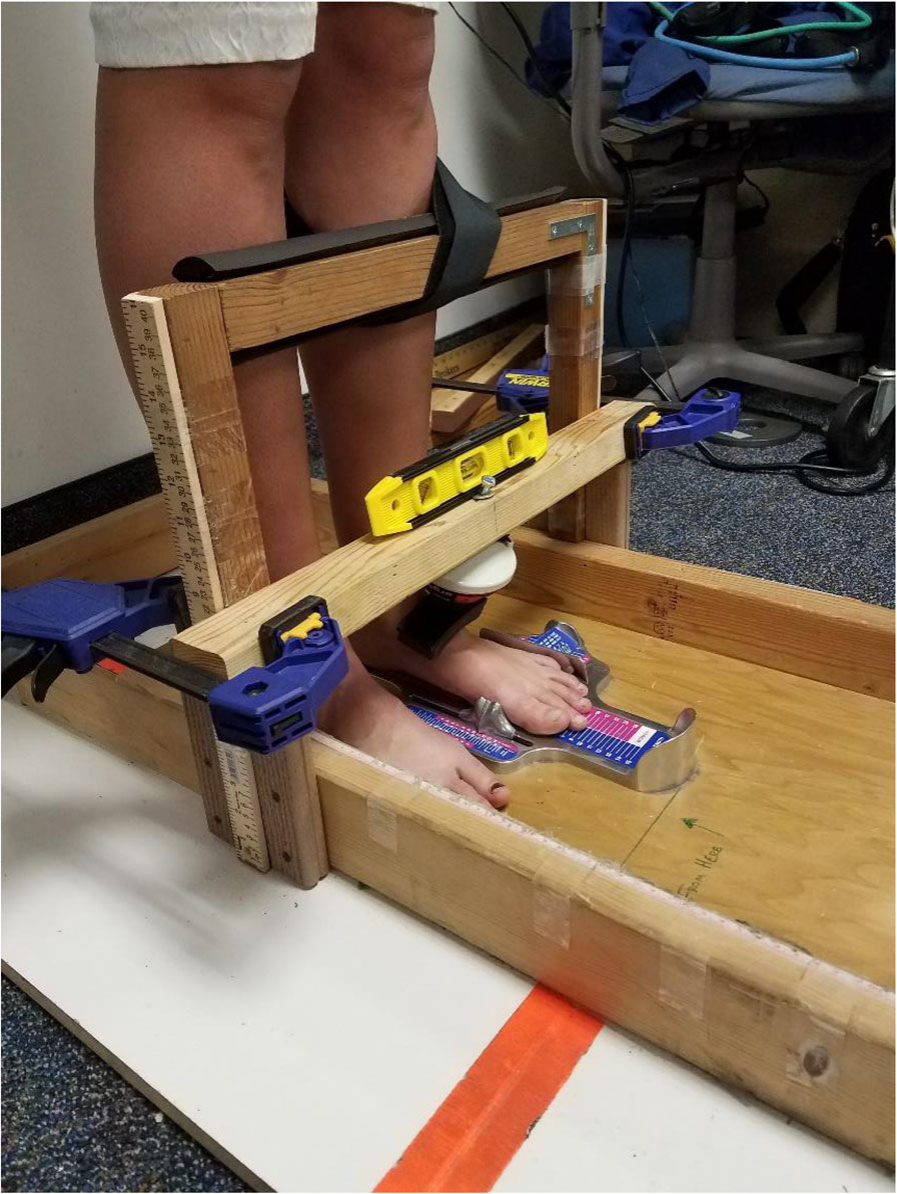
Set up for doming testing

Doming is performed by activating muscles to pull the metatarsal heads towards the heel of the foot, effectively shortening the foot (Fig. 2). In order to measure doming strength, participants stood with one foot in a Brannock device. The Brannock device was then moved anteriorly until the dorsum of the foot, just above the navicular tuberosity, rested against a handheld dynamometer (ErgoFet, Hoggan Health, Salt Lake City, UT) that was securely mounted to a wooden frame (Fig. 3). The dynamometer was calibrated with known weights and fitted to a trendline (r^2^ = 0.99). The anterior-posterior position of the Brannock device was adjusted so that the starting force was at 1 kgf. The subject then performed the doming action to a maximal voluntary contraction against the dynamometer for three seconds, then relaxed as instructed by the rater. Instructions for the doming action included, “keep your toes on the ground, slide the ball of your foot back towards your heel,” and “try to raise your arch without lifting or curling your toes.” Trials were repeated if the participant lifted their toes, the base of the first metatarsal, or the heel. To ensure this, a researcher was assigned to visualize the movement of the metatarsal heads.

**Fig. 4:**
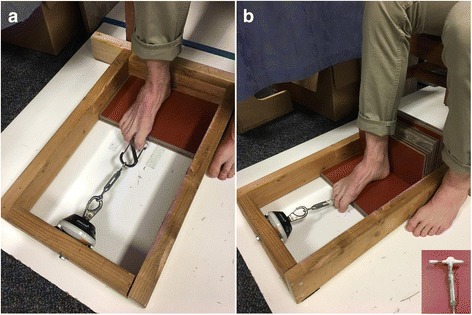
**a** Set up for hallux flexion testing, **b** Set up for lesser toes flexion testing. The inset image at the bottom right shows the T-bar that the subjects gripped during testing

Flexion strength of participant’s toes was assessed, first with the hallux unaided, then with the 2nd, 3rd, and 4th toes flexing simultaneously. During these tests, the dynamometer was attached to a wooden frame which was secured to the floor. Participants sat in a chair while researchers positioned the subjects’ knees to 90-degrees of flexion. Subjects placed one foot on an adjustable raised platform, with their heel against another set of panels (Fig. 4a). The panels behind the foot were interchanged depending on foot size so that the foot was supported from the heel to the head of the first metatarsal, while still allowing for unimpaired toe flexion.

To test hallux flexion strength, the foot was adjusted so that the hallux was aligned with the dynamometer. Then subjects gripped a carabiner attached to the dynamometer via a turnbuckle with their hallux. After adjusting the turnbuckle so that the toe produced a baseline force of 0.5 kgf with the toe at a 45° angle (Fig. 4a), participants were instructed to “flex the big toe and pull as hard as possible for three seconds, then relax your grip”.

The combined strength of each study participant’s 2nd, 3rd, and 4th toes was tested in a similar manner to their hallux, but instead of having a carabiner attached to the turnbuckle, a T-shaped metal bar was used. Subjects gripped the bar with the 2nd and 3rd toes straddling the metal piece that connected to the turnbuckle (Fig. 4b). A baseline force of 0.5 kgf was again established for each subject, and testing was carried out in an identical manner to the hallux flexion testing. Trials were repeated if the participant’s heel and/or ball of the foot were raised from the boards during the toe flexion tests.

Each test was repeated three times on each foot. Throughout each test, force data was collected at 100 Hz and recorded using ErgoFet Data Collection software. Peak force was subsequently determined using custom LabView software. An investigator visually inspected each force curve. In order to avoid false peaks, the peak force was determined as the highest point at which the force plateaued for more than 10 data points. The peak force value reported was calculated as the average of six data points surrounding the chosen point.

### Data analysis and statistics

Peak forces from three trials for each test performed during each session during both days of testing were used for statistical analysis. Interclass correlation coefficients (ICC_(2,k)_ and ICC_(3,k)_) were calculated in SPSS to test for reliability between raters during the same testing session on the same day (inter-rater, intra-day, intra-session), between raters on different days (interrater, inter-day, inter-session), between days for the same rater (intra-rater, inter-day, inter-session), and between sessions on the same day by the same rater (intra-rater, intra-day, inter-session).

## Results

ICCs showed good to excellent reliability for all tests (T1, T234, and doming) between days, raters, and sessions (Table 1) [20]. When tests were performed on the same day, the ICCs ranged from 0.71 to 0.93 for inter-rater reliability and 0.94 to 0.99 for inter-session reliability. The ICCs for testing performed on different days ranged from 0.90 to 0.95 for the same rater, and 0.80 to 0.82 for different raters. Results for ICC_(2,k)_ and ICC_(3,k)_ were numerically the same, as explained in Portnoy and Watkins (2009).

**Table 1:** ICC_(2,k)_ and ICC_(3,k)_ values for all comparisons

Inter-rater, Intra-day, Intra-session						
	Doming		Toe1		Toe234	
	ICC	95% CI	ICC	95% CI	ICC	95% CI
Rater 1	0.929	0.899 to 0.950	0.874	0.820 to 0.912	0.71	0.586 to 0.797
Rater 2						
Inter-rater, Inter-day, Inter-session						
	Doming		Toe1		Toe234	
	ICC	95% CI	ICC	95% CI	ICC	95% CI
Rater 1	0.816	0.760 to 0.858	0.803	0.733 to 0.855	0.802	0.731 to 0.854
Rater 2						
Intra-rater, Inter-day, Inter-session						
	Doming		Toe1		Toe234	
	ICC	95% CI	ICC	95% CI	ICC	95% CI
Day 1	0.949	0.927 to 0.966	0.903	0.861 to 0.936	0.924	0.890 to 0.950
Day 2						
Intra-rater, Intra-day, Inter-session						
	Doming		Toe1		Toe234	
	ICC	95% CI	ICC	95% CI	ICC	95% CI
Day 1	0.985	0.978 to 0.990	0.962	0.945 to 0.975	0.942	0.916 to 0.962
Day 2						

The values were the same for both variations of the ICC test. Where 95% confidence intervals differed, the lowest and highest values using both tests were included

Average doming strength was 99.96 ± 47.04 N. Average hallux flexion strength was 65.66 ± 24.5 N. Average lateral toe flexion was 50.96 ± 22.54 N. Average force values for each statistical analysis are included in Table 2.

**Table 2:** Average ± SD force values (in Newtons) for each test

Inter-rater, Intra-day, Intra-session			
	Doming	Toe1	Toe234
Rater 1	97.51 ± 47.92	64.88 ± 22.83	48.41 ± 17.35
Rater 2	100.94 ± 50.96	64.88 ± 23.52	52.63 ± 22.05
Inter-rater, Inter-day, Inter-session			
	Doming	Toe1	Toe234
Rater 1	92.96 ± 40.36	62.85 ± 22.98	48.08 ± 19.62
Rater 2	100.7 ± 38.61	70.28 ± 26.80	52.31 ± 25.73
Intra-rater, Inter-day, Inter-session			
	Doming	Toe1	Toe234
Day 1	90.85 ± 35.18	63.11 ± 19.11	50.67 ± 19.11
Day 2	99.86 ± 37.14	67.13 ± 22.83	53.51 ± 24.30
Intra-rater, Intra-day, Inter-session			
	Doming	Toe1	Toe234
Session 1	100.94 ± 54.19	62.92 ± 21.76	52.63 ± 19.01
Session 2	102.9 ± 53.31	60.86 ± 21.66	53.41 ± 20.68

## Discussion

The purpose of this study was to determine the reliability of novel methods of measuring muscle strength during doming and toe-flexion exercises. Inter- and intra-rater reliability were good to excellent for all tests. These results indicate that these methods of testing are repeatable among our rater group, which were moderately trained. The results of the ICC_(3,k)_ indicate that this repeatability can be expected from any group of raters who perform this testing. Therefore, these tests may be used for research and/or clinical purposes. The results from the inter-rater, inter-day and intra-rater, inter-day comparisons suggest, however, that for the most reliable comparisons, when a patient or research participant is tested multiple times, the same rater should perform the testing.

The doming strength measurements presented in this study represent a unique assessment that has not been analyzed before. Therefore, we are unable to compare our results to others. However, this is an important measurement to be able to quantify because this movement involves the recruitment of the tibialis anterior and posterior, flexor digitorum brevis, quadratus plantae, and abductor hallucis. These muscles have been shown to play an important role in arch support, the control of pronation, and postural control [13, 21]. Strengthening of the abductor hallucis has also been suggested as a treatment for hallux valgus [22], and pes planus [23]. Therefore, doming is a common exercise used by healthcare professionals in the treatment and rehabilitation of these and other foot pathologies. Having a reliable, non-invasive, and relatively cheap method of measuring the strength and function of muscles that support/lift the arch would allow clinicians and researchers to better monitor the effects of interventions (such as strengthening or orthotic use) and/or pathologies on the function of the foot. However, based on feedback from our subjects, doming is an unfamiliar movement and, for the most reliable results, it should be taught and practiced prior to the testing session for best results.

Average toe flexion strength values from the current study are comparable to, though often lower, than those from previous studies that employed dynamometry and tested toe flexor strength from the hallux and lesser toes separately. Direct comparisons between the current data and previously published data is difficult due to the differences in equipment and the positions used to test. With that said, the hallux flexion strength measurements reported here are very similar to the pre-intervention measurements in subjects with pes planus reported by Jung et al. [23] (62.2 ± 34.6 N and 62.5 ± 29.2 N). Meanwhile, our results are much lower than those reported by Spink et al. [1] (132.9 ± 31.1 N), possibly due to differences in positioning. Our tests were performed with the ankle in a neutral position. This was chosen because it is within the range of motion for common activities, such as standing, balancing, and walking. In this neutral position, both long and short flexor muscles contribute to the strength measured during toe flexion. In contrast, Spink et al. [1] tested subjects lying supine, with the ankle in plantarflexion and with a hand-held dynamometer placed at the interphalangeal joint of the hallux, which was in an extended position. This would alter the length-tension relationship and the force production of the muscles. Subjects were then instructed to flex the toe against the operator’s resistance. This may have allowed for increased contribution of the flexor hallucis longus when compared to our testing position, in which subjects started the test with the toe already flexed. Quek et al. [24] reported hallux flexor strength (79.58 ± 37.83 N), but the data was collected while subjects were standing rather than sitting. Spink et al. [1] were the only previous researchers to report flexion strength of the lesser toes in isolation from the hallux. Once again, our values are quite a bit smaller than those reported in that study (103 ± 27.5 N), likely for the same reasons previously discussed.

Many researchers have performed functional measures of foot muscle strength, though most test just the toe flexors. Some of these methods include qualitative testing, such as the Paper Grip Test, pushing against examiner resistance, or the Intrinsic Positive Test [1, 10, 16, 17, 25]. Our tests showed as good or better reliability than the Paper Grip Test and pushing against examiner resistance. No reliability has been reported for the Intrinsic Positive Test. In order to measure IFM strength and/or strength changes more accurately, various quantitative methods have been used as well. These include various uses of dynamometry, plantar pressure, and magnetic resonance imaging (MRI) [1, 5, 8, 15, 18, 19, 26–30]. While MRI may be considered a gold standard for measuring muscle size, it is not a direct measurement of muscle strength. In addition, MRI is expensive and difficult to obtain and analyze. Therefore, it is imperative that reliable quantitative methods of measuring functional foot muscle strength are developed. These quantitative methods have shown better reliability than the qualitative ones. The reliability of our measurements are similar to those reported from the aforementioned quantitative methods.

Quantitative strength testing methods vary by equipment used, as well as the number and action of the muscles tested. Studies that have reported foot muscle strength obtained via dynamometry have had subjects use all toes together [5, 15, 24, 29, 30], each toe separately [8], or (as in the current study) the hallux and a group of the lesser toes separately [1, 19]. Most of these tests were performed by having the subject push down on a bar or cuff with their toe(s) [5, 8, 15]. Others have had subjects grab a bar or cuff with their toes and pull into maximal flexion.

While the current data shows that the tests performed during this study are reliable, there are a few limitations that should be noted. Although our subjects reported that the carabiner and bar were comfortable, gripping and pulling with individual toes (particularly the lesser toes) may be an unfamiliar task, resulting in greater concentration on gripping the device than contracting the muscles. During the lateral toe flexion testing, most subjects naturally wanted to invert their foot to assist with the flexion movement. However, since the goal was to isolate flexion strength, the researchers made sure the foot was planted flat on the board during these tests, thereby limiting inversion. As previously stated, the testing positions were chosen because they placed the ankle in a neutral position, allowing for recruitment of the intrinsic and extrinsic muscles as they would be during a portion of common activities such as standing and walking. However, because of the small range of motion involved in these tests, these strength measurements may not be reflective of maximal activation during movements requiring larger ranges of motion and greater force, such as walking and running. Of the three tests performed for this study, the lesser toe flexion test was the most difficult to set up, which may explain the lower ICCs between raters. Getting the subject to grip the T-bar effectively, achieving the baseline threshold by adjusting the turnbuckle, and positioning the foot on the support boards are all potential sources of variability between raters. In addition, the lesser toe flexion tests were the last set of tests performed during each session. It is possible that subjects may have experienced some fatigue of the foot muscles.

## Conclusion

In conclusion, our three novel tests showed good reliability between testers and on repeated days of measurement. These are simple tests using relatively low cost equipment which could be used in a variety of situations to compare feet within a subject and/or monitor foot muscle strength changes in a population of interest.

## Abbreviations

cm: Centimeters
ICC: Intraclass correlation coefficient
IFM: Intrinsic foot muscles
kg: Kilograms
kgf: Kilograms of force
MRI: Magnetic resonance imaging
T1: Hallux flexion
T234: Flexion of the lesser toes
